# Quantifying cost of disease in livestock: a new metric for the Global Burden of Animal Diseases

**DOI:** 10.1016/S2542-5196(24)00047-0

**Published:** 2024-05-08

**Authors:** William Gilbert, Thomas L Marsh, Gemma Chaters, Wudu T Jemberu, Mieghan Bruce, Wilma Steeneveld, Joao S Afonso, Benjamin Huntington, Jonathan Rushton

**Affiliations:** a Institute of Infection, Veterinary, and Ecological Sciences, University of Liverpool, Liverpool, UK; b School of Economic Sciences and Paul G Allen School for Global Health, Washington State University, Pullman, WA, USA; c International Livestock Research Institute, Addis Ababa, Ethiopia; d College of Veterinary Medicine and Animal Sciences, University of Gondar, Gondar, Ethiopia; e School of Veterinary Medicine, Centre for Biosecurity and One Health, Murdoch University, Murdoch, WA, Australia; f Department of Population Health Sciences, Farm Animal Health section, Faculty of Veterinary Medicine, Utrecht University, Utrecht, Netherlands; g Pengwern Animal Health, Wallasey Village, UK

## Abstract

**Background:**

Increasing awareness of the environmental and public health impacts of expanding and intensifying animal-based food and farming systems creates discord, with the reliance of much of the world's population on animals for livelihoods and essential nutrition. Increasing the efficiency of food production through improved animal health has been identified as a step towards minimising these negative effects without compromising global food security. The Global Burden of Animal Diseases (GBADs) programme aims to provide data and analytical methods to support positive change in animal health across all livestock and aquaculture animal populations.

**Methods:**

In this study, we present a metric that begins the process of disease burden estimation by converting the physical consequences of disease on animal performance to farm-level costs of disease, and calculates a metric termed the Animal Health Loss Envelope (AHLE) via comparison between the status quo and a disease-free ideal. An example calculation of the AHLE metric for meat production from broiler chickens is provided.

**Findings:**

The AHLE presents the direct financial costs of disease at farm-level for all causes by estimating losses and expenditure in a given farming system. The general specification of the model measures productivity change at farm-level and provides an upper bound on productivity change in the absence of disease. On its own, it gives an indication of the scale of total disease cost at farm-level.

**Interpretation:**

The AHLE is an essential stepping stone within the GBADs programme because it connects the physical performance of animals in farming systems under different environmental and management conditions and different health states to farm economics. Moving forward, AHLE results will be an important step in calculating the wider monetary consequences of changes in animal health as part of the GBADs programme.

**Funding:**

Bill & Melinda Gates Foundation, the UK Foreign, Commonwealth and Development Office, EU Horizon 2020 Research and Innovation Programme.

## Introduction

Global demand for livestock and aquaculture products has been increasing for several decades, a trend that is projected to continue in line with increasing population, urbanisation, and wealth.^1^ On the supply side, increasing livestock populations and intensification of production have produced unintended effects on climate change, environmental degradation, and infectious disease epidemiology.^2^, ^3^

Most of the world's population remain reliant on animals for essential protein and micronutrients as part of a balanced diet;^4^, ^5^ additionally, a substantial proportion rely on animal power for transport and cultivation and for trade and income. Improving animal health therefore has been identified as a means to continue to meet societal demand for animals and their products and improve livelihoods, while alleviating some of the negative consequences of animal agriculture.^6^

Despite the enduring importance of livestock to society, the systematic collection and collation of data that would allow the valuation of animals and measurement of improvements in animal health over time are missing for many of the world's livestock and aquaculture production systems. As a means of addressing this deficit and drive positive change in global animal health, the Global Burden of Animal Diseases (GBADs) programme has been initiated.^7^

Through this programme, disease burden information is to be made available to smallholders and farmers, businesses, governments, and wider society as a global public good, following principles of transparency, inclusivity, and rigour. The aim of the programme is to foster an improved understanding of the contribution livestock (including aquaculture and wild animal hunting) makes to society, quantify the burden animal diseases place on society, and guide resources towards improvements in animal health leading to more efficient and sustainable production and food systems.

Farmed animals in terrestrial and aquaculture systems comprise a diverse group of species kept in systems that are similarly diverse in purpose, resources used, governance structures, and disease burden. Therefore, any method for measuring disease burden within the GBADs programme has to accommodate this diversity to be globally inclusive and consistent in its approach. Depending on stakeholder interest, results need to be presentable from the micro level, of the animal or farm, and to the macro level, of national and global populations. The huge variation in data availability across different animal species in different jurisdictions means the models used must, at this stage, be operative even with minimal input data. Finally, for consistency, the burden estimation method must consider and rectify the propensity for the inconsistent aggregation of single-cause or risk factor burden estimates that would result in overestimation of the total when summed together.^8^, ^9^

**Research in context**

**Evidence before this study**

In human health economics, the development of quality-of-life impact metrics, such as the disability-adjusted life-year, and the acceptance of standardised methods for disease burden estimation has provided analysts with the means to compare the burden of disease due to different causes in different populations. In livestock, where diseases have substantial economic repercussions, a unified procedure for measuring and expressing the burden of disease that allows comparative analysis across the diversity of global food animal species and food systems has been lacking until now. Previous studies have used heterogeneous methods to demonstrate the economic importance of specific diseases in livestock, the importance of livestock within the transmission of zoonotic diseases, and other external effects of livestock farming such as greenhouse gas emissions. The Global Burden of Animal Diseases (GBADs) programme is attempting to address the absence of standardised approaches to these issues, and this study was completed within the context of that programme. No systematic literature review was conducted as part of this study, instead, the method builds on established theoretical work on the cost of disease in livestock to develop a new metric.**Added value of this study** Although previous studies on the cost of disease in food animal species exist, they have focused on quantifying costs due to single causes with the implied risk of overestimation that this incurs. In this Article, we present a metric for the total cost of disease borne by livestock producers by measuring against a disease-free economically ideal health scenario. This metric, the Animal Health Loss Envelope (AHLE), produces an envelope into which all individual causes of cost should fit, and expresses the financial cost of changes in farm productivity due to disease. The ALHE is an important methodological step towards consistent estimation of disease costs across widely differing food animal species and production systems.

**Implications of all the available evidence**

Developing metrics for the standardised quantification of burden for animal diseases on society begins with the physical effects of disease within the economic context of the farm and the expenditure of producers trying to counter these effects. From here, the wider economic consequences across the food chain and society as a whole can be calculated. As the GBADs programme continues, the AHLE metric will allow the comparison of producers’ losses and expenditures across similar populations, with the potential to grant insight into the performance of markets for animal health services.

Proposing a method to meet this aim requires confronting substantial challenges. The methods that have been so successfully applied in the Global Burden of Disease study cannot be translated directly to animals, although many lessons can be learned. Human disease burden is most commonly measured through changes in longevity and quality of life in non-monetary units, such as disability-adjusted life-years, reflecting social and ethical concerns.^10^ However, agricultural animal populations are kept to serve an economic purpose with life quality and expectancy being subject to human management and market forces. Therefore, disease burden estimations in these animal populations have largely focused on converting changes in health state into monetary valuation in a manner more akin to cost-of-illness evaluation in humans.^11^ The GBADs programme follows this precedent and, where possible, uses monetary units to quantify disease costs on-farm, while recognising the need to connect the monetary costs of animal disease to other non-monetary costs, both on-farm and off-farm, to generate a total disease burden estimate.

Moving from conceptual to measurement models, here we present the first quantification of disease cost in the GBADs analytical framework, identifying a metric for estimating the costs of animal disease that are internal to the farm. From this approach, scenarios can be generated to feed into downstream models quantifying the wider monetary and non-monetary impacts of animal disease.

## Methods

### Definitions and concepts

Farmed animal populations are kept for an economic purpose, that of converting one set of resources into accessible nutrition or materials (eg, food), services such as traction or transport, or other goods, such as fibre or skins. An animal's ability to perform these functions can be compromised if it is in poor health. In biological terms, its ability to perform its economic purpose entails it, first, having sufficient energy and nutritional resources to maintain its physiology and perform the behaviours necessary for its own survival and, second, to then have sufficient surplus to yield whatever product is desired.

Disease in animals has been defined in the veterinary literature as “the abnormality of bodily structure or function”^12^ and “failure to produce at expected levels in the presence of normal levels of nutritional supply and environmental quality”.^13^ From these definitions, we propose a definition of a disease-free physical state to be a normality in bodily structure and function that enables the ability to perform physiological functions at normal levels, as long as nutrition and other environmental requirements are provided at adequate levels.

To narrow this general definition to the health of animals in a production process, Morris^14^ described the biological connection between health and productivity in livestock using the example of feed inputs. These effects, when generalised, lead to the conclusion that lost health influences the gross allocation of resources to the animal, the efficiency of their use per unit of product, the duration of productive life for each animal, and the rate at which the production process occurs over time. Bennett^15^ and Bennett and Ijpelaar^16^ disaggregated the direct costs of disease in livestock as being due to a reduction in output for a given level of input, a reduction in output quality, a higher level of input use, and additional expenditure on disease control. Bennett and Ijpelaar quantified the losses associated with 30 livestock diseases independently of one another by comparing the status quo with an attainable health state. However, as has been found for human diseases, handling causes of burden for individual diseases independently and summing to create a total can lead to overestimation.^17^ As such, substantial efforts have been made to produce demographic projections as a validity check against which disease burden estimates can be validated.^18^ The GBADs programme also needs to impose similar validity checks on total disease burden by estimating the total direct disease cost for all causes at farm-level via comparison with a disease-free ideal. This process can then be used to prevent overestimation when attributing costs to individual causes. Therefore, setting a clear definition of the ideal health state against which current production is to be compared is important to both the calculation and interpretation of this boundary.

### Quantifying the cost of health loss in animals

**Figure 1:**
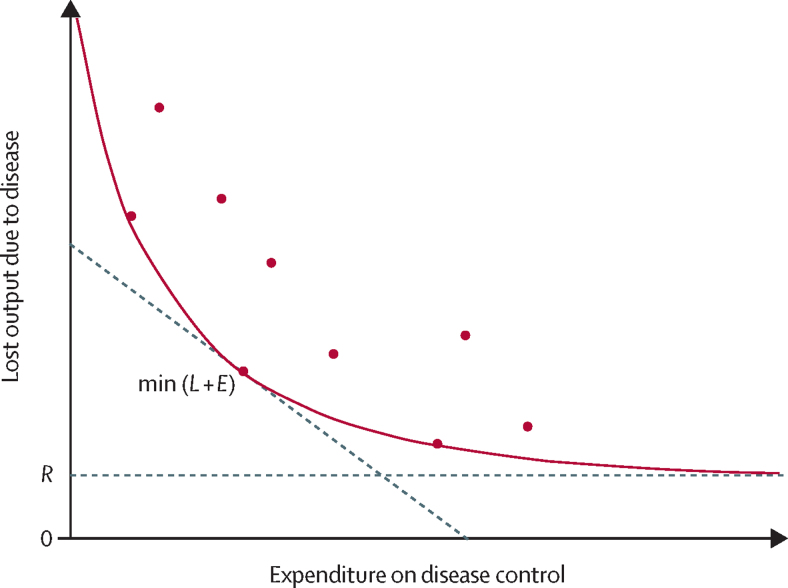
The loss-expenditure frontier due to disease in livestock production systems Adapted from McInerney et al.^19^ The figure illustrates the relationship between lost agricultural production due to disease and farm expenditure on disease control. If each point represents a single farm, the most efficient farmers are those who achieve the greatest reduction in loss for a given level of expenditure. These farmers mark out the efficiency frontier (the red curve). The economic optimum is the point on the frontier with the lowest combination of loss and expenditure marked min(*L* + *E*). The line at y=*R* is a residual level of loss that is beyond the capacity of current technology to mitigate.

These loss and expenditure components of disease cost have been described previously by McInerney and collaegues^19^ and updated by Tisdell^20^ to recognise the need to distinguish between different expenditure types. If the production losses and expenditure on disease control (eg, vaccination) for each firm in a population are mapped on opposing axes, the most efficient producers for each level of expenditure (ie, those that reach the greatest reduction in loss per unit of expenditure) can be identified and a frontier of technical efficiency^21^ for expenditure on disease control for the population mapped out (figure 1). The position of the frontier relative to the axes is dependent on the technologies to which the study population has access. The optimal combination of loss and expenditure is found at the point on the frontier where total cost if minimised, point min(*L* + *E*). McInerney and colleagues^19^ state that the purpose of generating information on the cost of disease is to allow action to take place and, given that the technical frontier and the area above comprise the zone of feasible change, this should also be where analysis takes place.

However, because of the technological limits on disease control—ie, diseases exist that cannot be perfectly controlled (ie, losses reduced to zero)—a residual exists (shown as *R* in figure 1), such that the frontier for all causes of loss will become asymptotic to the x axis as the limit of technological possibility is reached. The y-intercept of the frontier is the best case for zero expenditure and is dependent on the total exposure to disease faced by the population. Finally, the path of the frontier itself is dependent on the production function of the firms in the population, the genetics of the animals, and the inputs that are provided to them.

Therefore, using benchmarking or frontier analysis to estimate total disease cost in a single block becomes challenging. For each study population, the distance between the frontier and the zero-disease, zero-loss case at the origin is influenced by variation in the production process, and the physical and economic environment. To address this issue, the GBADs programme proposes that all-cause disease cost should be compared to a zero-cost ideal scenario, in which both the losses and expenditure due to disease are zero. This ideal health standard creates an envelope into which all disease burden on a farm should be enclosed. This concept for calculating disease costs against a zero-cost ideal health state has been termed the Animal Health Loss Envelope (AHLE).^22^ Presenting this method is the aim of this paper.

### Operationalising the AHLE concept

The objective of designing a measurement model for calculating the AHLE metric necessitates setting out a description of a generic livestock system functioning in both the presence and absence of disease. First, we present a conceptual model and, second, we discuss how to operationalise it.

The conceptual model follows the production–loss function approach of Hennessy and Marsh, as shown in equation 1:^23^

(1) $$y=\text{F}\left(z,\theta \right)\times \left[1-L\left(b_{0}\times \left(1-C\left(x,\theta \right)\right)\right)\right]$$

Here, a livestock system is described by an economic production function, where *y* is the output from the system and *F*(*z*,θ) is the production function for ordinary inputs (*z*) and exogenous parameters (θ; eg, climate and regulations). Ordinary inputs are the inputs (eg, animals, feed, and labour) that must be supplied in adequate quantity to generate *y*, even in the absence of disease. This conceptual model has antecedents in the crop-loss literature.^24^, ^25^ A loss function, *L*, which can take values between 0 and 1 inclusive, describes the action of disease-causing pathogens (*b*_0_; eg, hazards), on the production of *y. C*(*x*,θ), which can take values between 0 and 1 inclusive, is the control function, increasing in control inputs *x* (eg, vaccines and antibiotics, that mitigates the effects of *b*_0_). If *b*_0_=0, this is the disease-free case and there is no loss of output, such that *L*(0)=0. In those conditions, producers (eg, firms, households, farmers and livestock keepers) could adjust the ordinary input supply in light of improved efficiency, because ordinary input bundle (*z*) can be substitutes for disease control inputs, or because fewer resources are required to achieve the same objectives (eg, improved growth per unit of feed). In this condition, and without loss of generality for our purposes, the variable *R* in figure 1 would also be equal to 0.

Next, we turn to operationalising this model for the AHLE metric. Output under the ideal condition (or *L*(0)=0) is denoted as y*, wherein the production function reaches its full potential with the input amount, ideal input bundle z*, or y*=*F*(z*,θ). Therefore, the total disease costs are the sum of the change in value of control inputs *x*, output *y*, and ordinary inputs *z* between the current and the ideal conditions.

As already discussed and illustrated in equation 1, disease results in a rate change in output generated with respect to the inputs provided to the system. In livestock systems, the inputs used and outputs generated can be both numerous and contain joint interdependencies. We propose that a compartmental population model can be used to allow the consequences of simultaneous rate changes to be evaluated across a system with many degrees of freedom. Such models are well developed within the fields of ecology and epidemiology for modelling disease and other effects in populations.^26^, ^27^

The complexity of the system is reduced by assuming all input–output relationships are separable with respect to the individual animal, either by head or per unit of live mass, and that each compartment scales with constant returns on a changing population size. All rates of input use or output generation per animal unit are determined with respect to time and a standardised timeframe of a year is applied to make all rate changes comparable. Prices must then be brought in to turn this population model into an economic one.

To this end, each compartment within the population is numbered from 1 to *I*. The population of each compartment is then denoted as *n*_i_, where *i* takes values from 1 to *I.* Each ordinary input (*z*) provided to the population is listed and numbered from 1 to *K*. Each ordinary input quantity is denoted by *z*_k_, where *k* takes values from 1 to *K*. Disease control inputs are denoted as *x* and the quantity used in a compartment is given by *x*_i_*.*

To construct the output set, every live animal or product that exits from each compartment is included. This includes outputs for which there is demand (eg, live animals, meat, milk, or eggs), and those for which there is no demand or value (eg, dead animals and diseased carcases). Additionally the population of live animals at year start, t, and year end, t + 1, must be used to calculate a change in population for that compartment. This change is by convention listed as production output. Each output, *y*, can then be listed and numbered from 1 to *J*. Each output quantity is denoted by *y*_j_, where *j* takes values from 1 to *J*.

The live animal price in each compartment is then denoted by *s*_i_, the price of each output as *r*_j_ and the price of each ordinary input is *p*_k_. Disease control input price for a compartment is denoted as *q*_i_.

Supply alone does not determine outputs, but rather supply generates products to meet demand in a market system. If the demand quantity for each *y* is given by *y*^d^, the output constraint on the ideal health condition is that for each output, the output quantity in the ideal scenario (y*) must meet demand assuming constant pricing, as shown in equation 2:

(2) $$y_{j}^{*}\geq y_{j}^{d}$$

The following rules are used to define the population and production dynamics under an ideal health scenario. Rate changes for inputs used or output generated per animal head or per mass unit, with respect to time, can be provided where disease effects are removed, supported by available evidence. For mortality, a mortality rate of zero is assumed for each compartment. For the distribution of outputs generated by compartments, all disease-dependent exit routes are also set to zero rate (eg, unplanned culling, emergency slaughter, and condemnation of carcases). All rate adjustments must respect any environmental constraints (eg, legal and physical) that govern management. These external conditions are assumed to be constant between the ideal and current scenarios. Finally, control expenditure on disease in each compartment is also set to zero.

A cost minimisation optimisation is applied to the compartment population to determine the optimal compartment population termed *n*_i_*, which is the minimum number of animal units needed to satisfy equation 2 for all products.

With the ideal-health population size, input use, and output yield in hand, the AHLE metric (ideal production under zero disease presence and x*=0) can then be calculated as the total value difference across all compartments, in inputs used, control expenditure, and animal stocks, as the net of any additional output generated, as follows:

(3) AHLE=∑i=1I(∑k=1Kpk(zik-zik*)+qixi+si(ni-ni*)t-∑j=1Jrj(yij-yij*))

Equation 3 is equivalent to changes in profit (due to morbidity) and asset values (due to mortality) with prices held constant and, as such, is consistent with surplus measures of economic wellbeing for live animal producers.^23^, ^28^

### Role of the funding source

The funders of the study had no role in study design, data collection, data analysis, data interpretation, or writing of the report.

## Results

**Figure 2:**
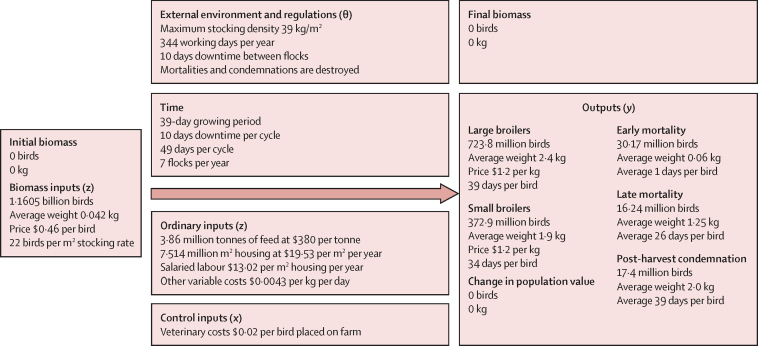
Flow diagram of broiler system performance in the current (with disease) scenario Currency is in 2020 US$. Averages are mean values.

To give a practical example of this calculation applied, a simplified model of intensive poultry production aimed at rearing birds for meat (ie, broiler chickens) is presented here. The deterministic spreadsheet model^29^, ^30^ (shown in the appendix) was used to estimate the population AHLE metric for a single year. The structure of the system is shown in figure 2.

First, the compartmental structure of the population is set. A single biomass input, the day-old chick, is grown and exits the compartment by one of several paths, thus a single compartment is required to describe the population. The demand set for the system, *Y*^d^, contains two outputs, large and small broilers, while the full output set contains three additional biomass outflows with zero value in the market, early mortalities, late mortalities, and post-harvest rejections. The system has an assumed population of zero birds at year start and end and therefore there is no change in population value within the output set. The ordinary input set *Z* contains the animal entry, the day-old chick, and four other items: feed, salaried labour, housing, and other variable costs. Given the stable supply of feed and water available to this population, the criteria of feed and water adequacy is assumed to be met. Any external constraints on production (θ) are defined. Current expenditure on veterinary inputs that comprise the control input set *X* is recorded per day-old chick entering the population. Finally, variable input–animal and animal–output relationships are defined for the current scenario by expressing each as per head or per kg of biomass. Bird-days and kg-days for animals and biomass, respectively, within the compartment are calculated to allow rates to be adjusted with respect to time.

To set the ideal scenario, the demand set for ideal health (*Y*^d^) contains large and small broiler yields at current quantities, and all mortality and condemnation is set to zero. These rate changes determine the necessary total placement of animals within the system across the 1-year period being modelled to meet demand at current prices. Rate changes based on animal performance per unit time are then applied to determine resource use across a disease-free system. The assumed change in performance between current and ideal health is given in table 1.

**Table 1:** Bird growth rate and body-mass–feed productivity under current and ideal health scenarios

	**Yield per head of population**		**Feed conversion ratio (kg feed per kg body-mass gain)**		Bird-days on farm per head*	
	Current	Ideal	Current	Ideal	Current	Ideal
Post-harvest rejection	0·015	0	1·60	1·34	37·3	35·3
Early mortality	0·026	0	0·22	0·20	1	1
Late mortality	0·014	0	1·31	1·20	26	26
Small broilers	0·32	0·34	1·49	1·33	34	32
Large broilers	0·62	0·66	1·60	1·41	39	36

* Post-harvest rejects are presented as a weighted average of small and large broilers, while each other category is presented as an integer value of days.

Changes in flock cycle length and chick placement rate are used to adjust the fixed-cost input requirements (ie, salaried labour, land, and housing) for the year. In the zero-mortality condition, stocking rates must be reduced to account for increased survivability and restrictions on liveweight density, which are commonly found in intensive broiler production systems.^31^ The stocking rate of the system, at 22 birds per m^2^ per cycle, is therefore adjusted to 21 birds per m^2^ per cycle. As the only disease control expenditure quantified, veterinary expenditure is set to zero per head of population in the ideal scenario. Total volume and value of ordinary and control inputs used by the system is then calculated. Other variable costs are scaled with kg-days of biomass (table 2).

**Table 2:** Change in expenditure between current and ideal health scenarios for AHLE estimation

	**Expenditure type**	**Expenditure, per year**	
		**Current**	**Ideal**
Day-old chicks	Ordinary	$538 035 988	$508 444 009
Labour, salaried	Ordinary	$97 824 725	$91 123 731
Land and housing	Ordinary	$146 737 088	$136 685 597
Feed	Ordinary	$1 491 827 059	$1 308 966 289
Veterinary costs	Control	$23 210 365	$0
Other variable costs	Ordinary	$196 895 267	$179 138 325
Total	NA	$2 494 530 492	$2 224 357 952

Data are in 2020 US$. ALHE=Animal Health Loss Envelope. NA=not applicable.

Following equation 3, the AHLE metric is then quantified as the net change in input costs (both ordinary and control), output generated, and biomass in the standing population between the two scenarios (table 3). The resulting cost of disease, at US$270 172 540, is approximately 9% of the total output value of the system under the current scenario, or approximately 11% of ordinary input costs under the current scenario being lost due to disease.

**Table 3:** AHLE for a national chicken meat production system over a 1-year period

	**Current**	**Ideal**	**Net**
Ordinary input cost	$2 471 320 127	$2 224 357 952	$246 962 175
Control input cost	$23 210 364	0	$23 210 365
Total output value	$2 934 741 755	$2 934 741 755	$0
Biomass valuation	$0	$0	$0
AHLE	NA	NA	$270 172 540

Data are in 2020 US$. ALHE=Animal Health Loss Envelope. NA=not applicable.

## Discussion

The AHLE extends established metrics of quantifying the costs of livestock disease against a zero-disease, zero-expenditure ideal standard. As such, it will be an integral part of the GBADs programme in which it will connect changes in the efficiency of livestock farming due to the effect of disease on animal biomass and resource use. It quantifies the financial cost to farmers of animal disease. Within the wider GBADs programme, development of this metric is a key step towards calculating estimates of the effect of changes in animal health on the economic wellbeing of society as a whole and the generation of negative environmental and public health externalities that are increasingly of global concern.

The AHLE metric connects the physical effects of morbidity and mortality to economic productivity of farms, but does not consider wider effects beyond the farm gate, or long-run adjustments in management due to changing incentives in the absence of disease. It presents the best-case scenario health outcome with current technology and management.

The need to place boundaries when attributing burden of disease to cause has already been described in the literature, where multiple data sources and the presence of comorbidities necessitate methods for, what can be termed, squeezing results within a defined total.^22^, ^32^, ^33^ The AHLE metric serves this purpose for GBADs disease burden estimates: when attributed to cause, the costs of the physical effects of disease and farm-level expenditure are quantified in a single farm-level measurement that cannot subsequently be exceeded by the total attributed to individual causes.

At a macro level, understanding the balance between losses to disease and expenditure, and the scope for moving the technical frontier with new technologies for a given population of livestock keepers can grant insight into the size and functioning of markets for animal health products. This information has been lacking in many regions globally, making the supply of veterinary products into those markets a challenge.^34^, ^35^

The use of a herd structural model based on multiple compartments, such as age and sex, where each compartment is also defined by inputs, outputs, and biomass inflow and outflow confers benefits from numerous perspectives. The effect of changes in fertility, growth, and culling rates on livestock populations often necessitates herd-structure modelling.^36^, ^37^, ^38^ Growth and product yields are usually associated with age and sex profiles, as can be disease incidence and mortality and morbidity rates. As a result, many livestock populations are readily disaggregated into groups of breeding, growing, and producing animals. Therefore, a compartmental population structure with age and weight profiles allows a more differentiated description of input and output flows to and from each compartment and provides a point for constraint in an ideal health scenario ensuring population stability.

Given the novelty of the zero-disease, zero-expenditure, ideal health scenario in livestock disease modelling and economics, a core concern of the GBADs programme going forward is the building of an evidence base to support parameterising these scenarios globally. Agronomists and climate scientists have advanced further in the domain of building global datasets and models on which to analyse yields and productivity.^39^, ^40^ As the GBADs programme establishes itself, it will aim to develop similar frameworks and methods to address the uncertainty that comes from attempting to measure an estimate from an unobservable ideal. Farrell^21^ expressed caution regarding use of a theoretical level of maximum technical efficiency in complex production processes, where this maximum might be used to analyse the performance of individual datapoints. Such concerns were noted during development of the ALHE. The AHLE is a metric that, in-of-itself, reflects an upper bound under ideal production and is not intended to be a practical benchmark or target to be aimed for by producers, but rather as a complement to existing performance benchmarking and as part of a suite of further metrics to illustrate how disease burden is distributed for live animal producers.

On its own, however, the AHLE metric only offers one view of the animal health situation in a given study population, because any effects outside of the farm are not captured, including price changes. For this reason, the AHLE metric will be complemented within GBADs by models of economic welfare and externality generation (eg, antimicrobial use, zoonotic disease, and greenhouse gas emission).

With biomass liveweight as a key input variable in the measurement model, the AHLE metric provides a means to connect biologically determined association, such as energy requirements and feed use, growth, and yields,^41^, ^42^, ^43^ with economic value through liveweight and output prices.^44^ Biomass also provides a denominator for productivity and resource use and, as opposed to head count, can be compared between species. For social wellbeing, the optimal number of animal units is key to balancing livestock inventories in a market supply and demand setting with mitigating environmental concerns. For the farm wellbeing, the cost-minimisation assumption is consistent with a profit-maximisation theme and, as such, allows the scenarios it generates to be aggregated to national and global levels.^28^

Biomass conversion factors for livestock species provide one means of connecting changes in population health to externalities that can have effects on the economy^45^ and society as a whole, such as climate change^46^ and antimicrobial use.^47^ Additionally, some global, regional,^48^ and national livestock statistics can be expressed in terms of biomass classifications and livestock units to better illustrate population structures and the value of economic activities and trade.

At present, the AHLE calculation method captures only a short-run adjustment of the system. Fixed-cost investments (eg, machines and infrastructure) and management salaries scale with the size of the animal population, which needs to be managed and housed. Fixed-cost and long-term investments that mitigate disease losses are not adjusted for savings in the absence of disease. Estimating these costs would require panel data that are not currently available globally, but that the GBADs programme plans to accumulate over the coming years.

At present a number of limitations exist that must be dealt with for the AHLE metric to become accepted as a measure of cost of disease. Perhaps most importantly, the uncertainty limits (or the credible interval) around animal productivity in ideal health must be addressed by GBADs to help users perform AHLE calculations. Global datasets facilitating analysis of genetic and environmental sources of variation in animal productivity are sparsely populated or inaccessible. These important population-level effects will need to be controlled for when calculating a globally complete set of AHLE estimates. Additionally, if the AHLE metric is interpreted out of context it could be misunderstood because, as already described, it calculates the farm-level cost of disease assuming constant prices. The metric makes no consideration for effects beyond the farm gate, including adjustments in resource and product prices and quantities used or produced as animal health improves. Therefore, the ALHE metric should be used as part of a suite of other metrics and analytical tools to place keep it within the appropriate context.

At the outset, however, disease cost is biologically at animal-level and economically at farm-level. Through the AHLE, the GBADs programme places farm-level costs of disease at the core of a linked system of disease burden metrics. The development of this metric provides insight into the relative contribution of output loss and expenditure on disease mitigation to the total financial cost of disease at farm-level. Going forward, this information will allow farm-level productivity change to be defined for quantifying the burden of animal diseases with respect to the wider economy and environment.

## Data sharing

A spreadsheet model illustrating the calculation method is available in the appendix. Further models and methods from GBADs are available at https://gbadske.org/. No other data will be shared.

For more on the **Global Burden of Disease study** see https://www.healthdata.org/research-analysis/gbd

## Declaration of interests

We declare no competing interests.
